# Audit on the appropriateness of integrated COPD management: the “ALT-BPCO” project

**DOI:** 10.1186/2049-6958-9-40

**Published:** 2014-07-18

**Authors:** Stefano Nardini, Gaetano Cicchitto, Fernando De Benedetto, Claudio F Donner, Mario Polverino, Claudio M Sanguinetti, Alberto Visconti

**Affiliations:** 1 Vice President/President Elect Interdisciplinary Association for Research in Lung Disease (AIMAR), Arona, NO, Italy; 2 Pulmonary Division Unit, Cava de’ Tirreni, SA, Italy; 3 AIMAR President, Arona, NO, Italy; 4 AIMAR Past President, Mondo Medico, Borgomanero, NO, Italy; 5 Secretary General/Treasurer AIMAR, High Specialty Provincial Pulmonologic Unit, “Scarlato” Hospital, Scafati, (SA), Italy; 6 Managing Director Multidisciplinary Respiratory Medicine, Rome, Italy; 7 Scientific Secretary AIMAR, Arona, NO, Italy

**Keywords:** Appropriateness, Assistance, Health organization, Specialized pulmonology practice

## Abstract

**Background:**

Non communicable chronic diseases (including respiratory ones) are the leading cause of death and disability. To cope with them we need to redesign the health system, improving primary prevention, screening, and outpatient services, while fully integrating different branches of the health service. The Italian Ministry of Health published extended guidelines on integrated COPD management (COPD-GL) in 2010. In2011 a condensed version was produced. These documents define appropriateness of management regarding both the specialist and the health service.

**Methods:**

An internal audit on how clinical practice conforms to COPD-GL standards was implemented in one Italian region involving 29 respiratory units (RU) (65.8% of the total regional RU): data were collected from the clinical database at time zero and after 6 months. In the meantime, specialists of RU underwent education on COPD-GL.

**Results:**

At time zero, significant gaps between current practice and recommendations emerged both in medical practice (mean agreement 25%) and in the health organization (48%). At month 6 the gaps were reduced more in clinical practice (60.7%) than in organization (54.7%).

**Conclusions:**

It is easier to resolve the gaps in specialist clinical practice than the organizational gaps, changing which is the politicians’ task. Correcting specialists’ inappropriateness may be worthless if this is not accompanied by improvement of the organizational obstacles. The search for appropriateness should not be limited only to specialists or to a strict control of drug prescription but should include all the organizational aspects. Implementation of COPD-GL calls for actions on the part of both specialists and the health system.

## Background

In September 2011 the United Nations (UN) stated that chronic non-communicable diseases (NCD) constitute a global emergency, in that they cause more deaths than all other causes combined [1]. Of the four considered to be the most important NCDs epidemiologically and economically speaking, one is chronic obstructive pulmonary disease (COPD) [2]. The official UN declaration as well as previous official documents of the World Health Organization (WHO) emphasized the importance of chronic lung diseases to such a point that in 2005 the Global Alliance against Respiratory Diseases (GARD) was set up by WHO, and officially launched in 2006 [3]. GARD, working through General Assemblies, has produced official recommendations and practical material for their implementation [4]. Also the European Respiratory Society (ERS) has produced a strategy document for the control of chronic respiratory diseases [5,6] which was presented on 6^th^ September 2011 at the European Parliament and, at the 2011 ERS Annual Congress. The Italian government, for its part, is a member of GARD International and has set up a national GARD [7] organization, aimed at building a network to improve the health care for chronic respiratory diseases.

All stakeholders involved agree on the need to modify the current model of health care for chronic disease management. The core of the new model must be prevention, both primary (universal and individualized) and secondary (early diagnosis), but not less important is the need to optimize the management of those already affected by the disease through continuity of treatment achieved by means of patient self-management, rehabilitation and the underlying supports required for palliative care [8].

AIMAR, which adhered to GARD when it was first set up in 2006, has tackled the issue of chronic respiratory diseases both from the perspective of education of specialists and general practitioners (GPs) and from the research point of view, setting up projects aimed at investigating specific aspects, such as for example the problem of underdiagnosis and the possibility of early diagnosis of obstructive respiratory disease through the project “SOS-Respiro” (SOS-Breath) [9].

The Italian National Health System is undergoing a period of profound restructuring in order to adapt to the changed epidemiological situation despite the severe restrictions imposed by the reduced availability of resources. For this reason, the challenge - more pressing in some regions of Italy than in others - is to achieve the changes envisaged by the national and international governmental organizations at an overall cost outlay that is lower than the current budget or, at least, that inverts the current trend for increase seen up till now.

In 2010 AGE.NA.S (National Agency for Regional Health Services) published the Italian guidelines for the management of COPD [10]. In the same year a joint-society Commission formed by the three major Scientific Societies operating in the field of respiratory medicine in Italy began producing – in conjunction with a scientific society of General Medicine – a document to translate these guidelines into practice, creating a “light”, practical, “user-friendly” product by means of schemes and flow diagrams. This document was published in 2011 [11], and was officially presented to the Senate of the Republic the same year and an updated version followed in 2013 [12]. Immediately following the production of these documents, the need was felt to verify if and to what extent their implementation was possible in clinical practice and, if difficulties were found, the reasons for these problems.

AIMAR, which took part with its representatives in the drafting of both the above documents, launched back in 2011 the editorial project ALT-BPCO “Appropriateness of Long Term treatment of COPD” which had as its objective to verify the applicability of the guidelines and evidence eventual obstacles for their implementation. ALT-BPCO (STOP-COPD in English) was launched as a pilot project in one Italian region with the aim to provide health professionals with the up-to-date elements to reflect on their own diagnostic and therapeutic habits and help them guide and manage the organizational changes.

This article reports the results of the above audit project.

## Methods

The audit consisted in comparing the clinical reality with a defined standard in order to identify significant gaps and propose consequent actions for improvement. In an Italian region, Campania, a group of specialists from 29 Pulmonology Units, representing about two-thirds (65.8%) of the Pulmonology Units present in the region, agreed to take part in the project scheduled to be carried out in 2012. Their role was to compare the data contained in their own outpatient records regarding patients affected with COPD (“the clinical reality”) with the current recommendations (“the standard”). The study design consisted in a preliminary set-up phase, a first investigative phase followed by a meeting to discuss findings (in which all Centers were involved) and a second phase of survey and verification, then a final meeting to compare findings, verify what gaps existed and present the study results.

In the preliminary phase, the scientific committee established the instruments for the survey, i.e. the data collection forms. It took as the starting point the recommendations of the guidelines and joint-society document [10,11] in the field of prevention, early diagnosis and care. Three different forms were created. First, an Audit form, which was used to record the clinical and functional data extracted from the outpatient records of patients (50 per Pulmonology Unit) diagnosed with COPD, in order to evaluate their conformity with what is established by the diagnostic and treatment standards. These audit forms were in the possession of each Pulmonology Unit. A second form, a Summary Outline of the audit forms, was used to summarize the results of the 50 audit forms in a single report, completely anonymous, which was sent to the Scientific Society. A third form, also sent to the Scientific Society, the Implementation form, contained the responses of individual specialists to questions aimed at verifying the effective availability at organizational level (e.g. setting up of the network) of what the guidelines recommend.

In a first plenary session held in February 2012 the data collection forms were shared, analyzed and discussed with all participants.

Concerning the Audit form, the standard on which it was based (“benchmark”) were the recommendations of the AGE.NA.S. guidelines [10] in relation to the appropriate diagnostic classification and staging and the Joint society document [11] for the successive follow up of the COPD patient. The Patient form thus contained all the parameters recommended in these documents, as reported in Table 1 (alongside each parameter is reported the reference to one of the two documents and relative page number).

**Table 1:** **Parameters included in the Audit form** (for an explanation, see text [10,11])

1	Spirometry performed less than 1 year previously ( [10], pg. 38)
2	At least one global spirometry ( [10], pg. 40)
3	Functional severity stage ( [10], pg. 44)
4	Body mass index (BMI) ( [10], pg. 46)
5	Comorbidities ( [10], pg. 155-160)
6	Dyspnea ( [10], pg. 46)
7	Type of scale used to assess Dyspnea ( [10], pg. 46)
8	Walking test ( [10], pg. 41)
9	Blood oxygen saturation ( [10], pg. 40)
10	Arterial blood gases analysis ( [10], pg. 40)
11	Smoking status ( [10], pg. 54)
12	Pharmacological and behavioral smoking cessation treatment for smoker patients ( [10], pg. 195)
13	Number of exacerbations in the previous year ( [11], pg. 26)
14	Number of exacerbations treated with antibiotic and systemic steroids in the previous year ( [11], pg. 26)
15	Treatment with oxygen therapy ( [10], pg. 132)
16	Hospital admissions for COPD in the previous year ( [10], pg. 131)
17	Admissions with use of mechanical ventilation ( [10], pg. 133)
18	Second admission in less than three months from the previous hospitalization
19	Prescribed long-term respiratory therapy ( [10], pg. 58-80)
20	Prescribed drugs ( [10], pg. 58-80)
21	Need for pulmonary rehabilitation ( [10], pg. 168)
22	Setting of pulmonary rehabilitation

Concerning the Implementation form, the study requested participants to report the data indicated in Table 2. Also in this case, the standards (“benchmarks”) were those of the AGE.NA.S. guidelines [10] and Joint society document [11]. Also in Table 2 alongside each parameter is reported the reference to one of the two documents and relative page number. Some parameters (e.g. n. 2, 5, 12) were included in that they were implicit in the recommendation to form a “network” of health professionals ( [11], pg. 31 and following).

**Table 2:** **Data to be recorded in the Implementation form (for an explanation, see text**[10,11]**)**

1	Method of assessing the presence of bronchial obstruction ( [10], pg. 38)
2	Organization of meetings with the reference GPs to exchange information in the year preceding the survey
3	The possibility to administer, through the GPs in their area, screening questionnaires for COPD ( [10], pg. 44)
4	Knowledge about the risk cards for COPD of the National Health Institute (NIH) ( [10], pg. 43)
5	The possibility to use, with GPs of their area, the risk cards
6	The possibility to provide pharmacological and behavioral therapy to COPD smoker patients ( [10], pg. 57)
7	The modes of prescription of long-term oxygen therapy (LTOT) at home ( [10], pg. 84)
8	Periodic verification of the indication for and effective use of LTOT ( [10], pg. 84)
9	The effective possibility to offer patients pulmonary rehabilitation treatment ( [10], pg. 178)
10	The availability of care facilities for COPD patients in the acute phase ( [10], pg. 87)
11	The possibility to educate patients as regards self-management ( [10], pg. 178)
12	The possibility to jointly agree with the patient’s GP on discharge of hospitalized COPD patients
13	The availability of specialist home care ( [11], pg. 3-32)
14	The availability of tele-care facilities ( [11], pg. 32)

It was arbitrarily decided that, if the parameter recommended by the guidelines as necessary to ensure appropriateness of the service was present in more than 80% of the patient records examined (parameters reported in the Audit forms and then transmitted in the Summary forms), the correspondence and thus the clinical appropriateness was considered as excellent*.* For lower levels of agreement, the parameters were ascribed a value arbitrarily based on the score, as shown in Table 3.

**Table 3:** Scoring and evaluation of appropriateness in relation to the standards

**Score**	**Evaluation**
> 80%	Excellent
60–80%	Good
40–59%	Adequate
20–39%	Inadequate
< 20%	Very inadequate

Concerning, on the other hand, appropriateness of care at the organizational level (Implementation form) it was decided that this was not possible if the elements considered were absent, and thus the evaluation given was established as adequate/inadequate based on whether the score was, respectively, higher or lower than 80%. This criterion may appear excessively “generous”, but the Scientific Committee felt constrained to establish this cutoff since to date standardized or valid criteria have not existed for the management of COPD, which has been seen as the responsibility of the specialist as an individual rather than as part of a well-defined diagnostic**-**therapeutic system.

In the following months (March-April 2012), data collection was carried out, in each Center, followed by the elaboration of the data transmitted to the Scientific Society (first phase – first survey). In the month of May a meeting of the study group was held to analyze and discuss these results. Education on the guidelines was provided, which covered the key difficulties in their implementation evidenced by the first phase of data collection. Then, in the month of October the data collection and successive elaboration was repeated (second phase), followed by a comparison of the results between the two survey phases.

## Results

Concerning the Audit forms (specialist practice), the overall sample surveyed consisted of 1,450 patients. The results of the analysis of the Audit forms are summarized in Table 4. Concerning the Implementation form, the study sample consisted of 29 centers. The findings of the analysis of the Implementation forms are summarized in Table 5. In Tables 4 and 5 the data compared, obtained in the two phases of the project, are presented graphically as follows: first phase/*second phase.*

**Table 4:** Summary forms

	First phase/*second phase*									
NUMBER OF CENTERS INVOLVED:	29/*29*									
NUMBER OF PATIENTS ANALYZED IN EACH CENTER:	50/*50*									
TOTAL PATIENTS ANALYZED:	1,450/*1,450*									
*NOTE: THE MEAN VALUES REPORTED BELOW REFER - UNLESS OTHERWISE INDICATED - TO THE PRESENCE OF THE PARAMETER EXPRESSED AS A PERCENTAGE OF THE TOTAL (IN PRACTICE EACH CENTER TRANSMITTED THE PERCENTAGE OF THE TOTAL OF ITS 50 PATIENTS AND THE RESULT REPORTED HERE REPRESENTS THE SUM OF THE PERCENTAGES OF ALL CENTERS).*										
Mean% of COPD patients with record of a spirometry test performed less than 1 year ago	87,31/*91,52*									
Mean% of patients reporting global spirometry	30,75/*45,81*									
Mean% of patients with at least one global spirometry reported	42,52/*50,99*									
Mean% of patients with reported record of functional severity staging	81,82/*91,45*									
Mean n. of patients by level of severity (out of total patients of the 29 centers.	MILD	MODERATE	SEVERE	VERY SEVERE	*NOT INDICATED*					
	8,31/*10,12*	15,86*/16,1*	12,05/*15,01*	8,32/*9,24*	5,96/*0*					
Mean% of patients with reported BMI data	66,13/*68,86*									
Mean% of patients with reported presence of comorbidities	74,58/*88,79*									
Mean% of patients with reported presence di dyspnea	46,65/*59,21*									
Scale used for the assessment of Dyspnea (NOTE: number of centers per type of scale):										
MRC	8/*10*									
VAS	0/*0*									
BORG	2/*5*									
OTHER	1/*0*									
VAS + BORG	6/*2*									
MRC + VAS + BORG	3/*3*									
MRC + VAS	1/*1*									
MRC + BORG	3/*7*									
*NO RESPONSE*	5/*1*									
Total	29/*29*									
Mean% of patients with 6-min walking test reported	30,96/*37,55*									
Mean% of patients with a SatO_2_ reported	74,62/*90,41*									
Mean% of patients with a blood gases analysis reported	40,96/*53,1*									
Mean% of patients with smoking status reported	75,20/*85,1*									
Mean% of smoker patients treated with behavioral smoking cessation therapy	9,79/*14,1*									
Mean% of patients with report of eventual exacerbations in the previous year	47,89/*63,59*									
Mean n. of patients with n. exacerbations (out of total patients of the 29 centers).	0	1	2	3	MORE THAN 3	*NOT INDICATED*				
	6,13/*10,96*	7,56/*20,05*	5,22/*7,37*	1,55/*2,5*	3,7/*8,54*	25,66/*0*				
Mean% of patients with exacerbations treated with antibiotic and systemic steroid	45,03/*62,26*									
Mean% of patients on home oxygen therapy	25,24/*36,59*									
Mean% of patients with hospital admissions for COPD reported in the preceding year	28,67/*44,9*									
Mean n. of patients with n. admissions (out of the total patients of the 29 centers).	0	1	2	3	MORE THAN 3	*NOT INDICATED*				
	6,37/1*2,76*	4,11/*10,8*	2,05/*5,98*	0,76/*4,72*	1,03/*15,16*	*35,68/0*				
Mean% of patients with use of mechanical ventilation reported	7,36/*22,59*									
Mean% of patients with report of a second hospital admission within a short delay (max 3 months) from the first	7,62/*9,48*									
Mean% of patients with long-term respiratory therapy prescribed	80,93/*85,93*									
Prescribed therapy (N. centers that indicated the specific therapy; N.B. most Specialists/Centers indicated more than one therapy)										
SABA AND/OR LAMA	LABA	ULTRA LABA	ULTRA LABA + LAMA	LAMA	LABA + LAMA	FIXED COMBINATIONS LABA + ICS	XANTHINE	PDE4 INHIBITORS	OTHER THERAPY	NO THERAPY INDICATED
10/*11*	11/*21*	14/*20*	18/*20*	20/*25*	13/*17*	20/*29*	13/*12*	14/*17*	3/*11*	1/*0*
Mean% of patients with need for pulmonary rehabilitation (PR) recorded	23,10/*25,55*									
Place where data sheet was carried out (n. centers per place of identification):										
HOME + DAY HOSPITAL	1/*0*									
NO RESPONSE	8/*7*									
DAY HOSPITAL	4/*4*									
HOME	4/*7*									
INPATIENT (IN NON-ACUTE WARD)	5/*6*									
DAY HOSPITAL + INPATIENT (IN NON-ACUTE WARD)	2/*0*									
OUTPATIENT	5/*5*									

**Table 5:** Implementation forms

**METHOD USED BY THE CENTERS FOR ASSESSMENT OF BRONCHIAL OBSTRUCTION (First phase/ *second phase *)**			
	FIXED RATIO FEV1/FVC N^		25 Centers (86,2%)/*25 Centers (86,2%*)
	LOWER LIMIT OF NORMALITY (LLN)		4 Centers (13,8%)/*4 Centers (13,8%*)
**CENTERS THAT HAD RECENTLY (<1 YEAR AGO) AT LEAST ONE EXCHANGE WITH GPs ON METHOD FOR CLASSIFYING RESPIRATORY SYMPTOMS**			
	YES		23 Centers (79,3%)/*25 Centers (86,21%)*
	NO		6 Centers (20,7%)/*4 Centers (13,79%)*
**CENTERS THAT RETAIN THE APPLICATION POSSIBLE, THROUGH THEIR LOCAL GPs, OF A SCREENING QUESTIONNAIRE FOR COPD SYMPTOMS**			
	YES		28 Centers (96,6%)/*29 Centers (100%)*
	NO		1 Center (3,4%)/*0 Centers (0%)*
**CENTERS AWARE ABOUT THE NHI REPSIRATORY RISK CARDS**			
	YES		26 Centers (89,7%)/*28 Centers (96,55%)*
	NO		3 Centers (10,3%)/*1 Center (3,45%)*
**CENTERS THAT CONSIDER THE NHI RISK CARDS APPLICABLE BY THEIR LOCAL GPs**			
	YES		22 Centers (75,9%)/*22 Centers (75,9%)*
	NO		7 Centers (24,1%)/*7 Centers (24,1%)*
**CENTERS THAT PROVIDE, DIRECTLY OR THROUGH A DEDICATED SERVICE, THEIR COPD SMOKER PATIENTS WITH BEHAVIORAL-PHARMACOLOGICAL SMOKING CESSATION TREATMENT**			
	YES		21 Centers (72,4%)/*21 Centers (72,4%)*
	NO		8 Centers (27,6%)/*8 Centers (27,6%)*
**METHOD USED BY THE CENTERS FOR PRESCRIBING HOME LONG-TERM OXYGEN THERAPY**			
	PRESCRIPTION BY A SPECIALIST OR CONFIRMED BY A SPECIALIST IF MADE BY A NON PNEUMOLOGIST		20 Centers *(69,0%)*
	OTHER (THE ONLY PRESCRIBING CENTERS IN THE HOSPITAL SYSTEM)		9 Centers (31,0%)
	ANYONE CAN PRESCRIBE O_2_ WITHOUT NEED FOR CONFIRMATION BY A PNEUMOLOGIST		0 Centers (0%)
*(N.B. IN THE SECOND PHASE THE RESPONSE OPTIONS WERE DIFFERENT)*			
	*PRESCRIPTION BY A SPECIALIST OR SPECIALIST CONFIRMATION NEEDED IF PRESCRIPTION MADE BY A NON PNEUMOLOGIST PHYSICIAN*		*20 Centers (69,0%)*
	*ONLY THROUGH PRESCRIPTION BY A SPECIALIST*		*1 Center (3,44%)*
	*ONLY THROUGH PRESCRIPTION BY A HOSPITAL-BASED PNEUMOLOGIST*		*1 Center (3,44%)*
	*NO RESPONSE*		*1 Center (3,44%)*
	*OTHER*		*6 Centers (20,68%)*
**CENTERS THAT PERIODICALLY CHECK THE OXYGEN FLOW PRESCRIBED AND IF THERE IS STILL NEED FOR LTOT**			
	YES		27 Centers (93,1%)/*27 Centers (93,1%*)
	NO		2 Centers (6,9%)/*2 Centers (6,9%*)
**CENTERS THAT ARE EQUIPPED TO PROVIDE, DIRECTLY OR BY MEANS OF A DEDICATED SERVICE, PULMONARY REHABILITATION TREATMENT TO THEIR COPD PATIENTS**			
	YES		13 Centers (44,8%)/*17 Centers (58,62%)*
	NO		16 Centers (55,2%)/*12 Centers (41,37%)*
**CENTERS THAT ARE EQUIPPED TO TREAT, DIRECTLY OR BY MEANS OF A DEDICATED SERVICE, THEIR COPD PATIENTS IN ACUTE RESPIRATORY FAILURE, THROUGH A:**			
	UNIT OF INTENSIVE INTERMEDIATE RESPIRATORY CARE		5 Centers (17,2%)/*7 Centers (24,13%*)
	FOLLOW UP UNIT		9 Centers (31,8%)/2 Centers (6,89%)
	INTENSIVE CARE UNIT		6 Centers (20,7%)/*5 Centers (17,24%)*
	INTENSIVE CARE UNIT + UNIT OF INTENSIVE INTERMEDIATE RESPIRATORY CARE		1 Center (3,4%)/*8 Centers (27,58%)*
	NO RESPONSE		8 Centers (27,6%)/*7 Centers (24,13%)*
**IS YOUR CENTER ABLE TO OFFER PATIENTS A COURSE OF EDUCATION ABOUT THE DISEASE?**			
	YES		13 Centers (44,8%)/*14 Centers (48,27%)*
	NO		16 Centers (55,2%)/*15 Centers (51,72%)*
**CENTERS ORGANIZED TO CONSULT WITH THE GP REGARDING THE PATIENT’S DISCHARGE FROM HOSPITAL**			
	YES		7 (24,1%)/*8 Centers (27,58%)*
	NO		22 (75,9%)/*21 Centers (72,41%)*
**CENTERS EQUIPPED WITH A HOME CARE SERVICE**			
	NO SERVICE AVAILABLE		12 Centers (41,4%)/*16 Centers (55,17%*)
	SAME FOR ALL CHRONIC PATIENTS IN HOME CARE WITH DISTRICT NURSES		17 Centers (58,6%)/*12 Centers (41,37%)*
	SPECIALIZED FOR CHRONIC RESPIRATORY FAILURE PATIENTS		0 Centers (0%)/*1 Center (3,44%)*
**CENTERS EQUIPPED WITH A TELE-ASSISTANCE SERVICE**			
	YES		0 Centers (0%)/*0 Centers (0%)*
	NO		29 Centers (100%)/*29 Centers (100%)*

## Discussion

In Italy the current levels of care for people affected by COPD consist of emergency or, in any case, non-programmed hospital (outpatient or inpatient) interventions for acute events (real or perceived as such by the patient), but these interventions are not part of an organic structure or follow up system organized according to the principles of “managed care” or integrated care [13,14], such as is already in practice elsewhere for COPD with positive results [15].

It should be stressed that in any chronic disease, and so in COPD too the goal to strive towards is an “integrated” approach [8] based on the fundamentals of self-management by the patient and caregiver through simple instruments of control, lifestyle modification, long-term treatment, and rehabilitation. For Pulmonology the hallmarks are patient education about the disease (its nature and course, as well as the signs and symptoms of its worsening), smoking cessation, optimal bronchodilation and exercise training. In the advanced stages, oxygen therapy and home ventilation therapy come to play a fundamental role followed, in the terminal stages, by end-of-life care. A recent document of the American Thoracic Society [16] emphasizes that optimal treatment of COPD patients calls for an individualized patient-centered approach that addresses all aspects of the disease, manages the systemic effects and comorbidities, and integrates the medical treatment both inter-professionally (amongst the different health professionals) and inter-sectorially.

Translating this into daily pulmonology clinical practice, the key concept on which integrated care hinges is clearly appropriateness**,** i.e. an “appropriate” use of the diagnostic tools, drugs, physical therapies and rehabilitation procedures which should be made by the most “appropriate” professional figure at a given stage of the disease’s natural history, with an “appropriate” use of economic resources.

To use the words contained in the Joint-society document for the dissemination of the AGE.NA.S guidelines [11]: *“…the best management of the COPD patient is achieved through integration between the Pulmonologist, GP and other specialists, involved each in turn as their expertise is required; for each professional figure the tasks to perform must be well defined, in the context of a common diagnostic and therapeutic path that is appropriate for the different levels of disease severity.*” And since it is in the interests of both the patient and the general public that COPD does not evolve towards stages of greater severity or that this is delayed as long as possible, it is necessary to eliminate “*the main risk factors and install a treatment that is pharmacological and non pharmacological, adequate and continuing in time, and diversified according to the level of disease severity*”.

The instruments for updating knowledge and integration are certainly the guidelines and the diagnostic-therapeutic processes, in particular the latter because they enable each professional figure to understand his/her own place in the care pipeline and the relative figures of reference further up or down the pipeline. But it is just as important, given the need to know the patient’s situation up-to-date (practically, in ‘real time’), to have logistic-operational supports available for the routine networking of information (in particular, patient medical charts designed according to the guideline standards, so that information contained in the GP’s records or in an online patient medical file is instantly available to all involved), the possibility of home specialist care, able to be activated at any moment, and the availability of pulmonary rehabilitation. Without these supports, the realization of which is beyond the capacity of the single health professional, it is not possible to make any evaluation of appropriateness: an appropriate therapy at the time of the exacerbation could become excessive (and hence inappropriate) once the acute event has passed. Oxygen therapy, indispensable at the time of the exacerbation, could become superfluous (and uselessly expensive) or even dangerous once the patient has returned to the state prior to the acute event. In pulmonology very often care continuity and access to the latest information about the patient are lacking and so the therapy continues without being updated (and thus is at risk of being inappropriate), until the next exacerbation.

The aim of the editorial project ALT-BPCO was to measure right from the outset how well individual health professionals, their specialist wards, as well as the larger operational context conform to the management recommendations stemming from the AGE.NA.S guidelines [10] and their updated disseminated version [11] designed to facilitate their implementation. The data collection forms made it possible to verify the adequacy of the overall performance both of the specialist discipline and of the organizational context in which it is inserted. The strong and weak points of specialist activity, as well as of the context, were “photographed” respectively through the Summary and Implementation forms.

At the level of lifestyle changes, the “photo” of the situation regarding smoking cessation shows what the current weak points are. The guidelines state: *“…in this context, smoking cessation is, on the part of the patient, categorical*” and: “.*..all smoker patients diagnosed with COPD, in whom smoking cessation is an essential therapeutic measure, must be provided with behavioral and pharmacological treatment for the cessation of the smoking habit…”.* Patients in whom smoking status was recorded (i.e. smoker yes/no?) represented only 36.05% of the total at the first survey, a result that is clearly inadequate. At the second survey this percentage rose to 85%, which was defined as excellent. Recording of the use of pharmacological and behavioral treatment to quit smoking resulted at the first assessment to be highly inadequate (3.44%), and this score – though improved – remained still inadequate at the second assessment (24%). Finally, only 21 out of 29 centers (72%) are equipped to provide COPD smoker patients with behavioral-pharmacological treatments to quit smoking. As predictable, this percentage remained unvaried at the second survey; in other words, although the specialists tried – with success – to improve their performance, the context in which they operate – which was also inadequate – did not change. From these findings one can conclude that smoking treatment was not made available to all who needed it and this is attributable both to the incomplete professional training, and to the insufficient availability (on the part of the National Health System) of specialized personnel for smoking cessation.

As regards early diagnosis, this is made possible by patients’ recognition of their own symptoms – which are present but often ignored or underestimated by the patient – as well as by their awareness of belonging to a high risk class. The situation analyzed through our sample seems positive given the recorded possibility of using a questionnaire that focuses the patient’s attention on their own symptoms (97% and 100%), even if obviously it is necessary to clarify the “setting” in which such a questionnaire can be used.

The risk cards produced by the NHI, utilizable for the screening of the general population, are well known (by almost all, at the second survey), but are held to be not universally applicable (by 22 centers out of 29, 76%, which remained unvaried at the second survey). This seems to be attributable to the fact that while the first instrument (questionnaire) is filled out by the patient, the second (risk cards) requires a greater intervention on the part of the GP, whose time is already heavily burdened.

The appropriateness of therapy, once the diagnosis of bronchial obstruction has been formulated, refers both to the functional level and to the symptomatology of the patient [10,11]. As far as the diagnosis is concerned, 86% of the sample examined uses the so-called fixed ratio (FEV_1_/FVC < 70%) and only 4 out of 29 centers use the lower limit of normality (LLN). It seems to us that this result cannot be considered a sign of diagnostic inadequacy (and consequent therapeutic inappropriateness) because, even if there is evidence in the literature demonstrating overestimation of the disease in males and in elderly persons [17], it is also true that – as underlined in the AGE.NA.S guidelines [10] - “*the current lack of reliable estimates of the distribution of values of the FEV*_*1*_*/FVC ratio in the various age-groups renders impracticable a diagnosis based on values below the 5*^*th*^*centile of the distribution of FEV*_*1*_*/FVC in the reference population, considering that the 5*^*th*^*centile is chosen conventionally as the lower limit of normal values.”* Moreover, given that spirometry is not carried out on the whole population but only on those at risk or symptomatic individuals, for whom there is thus a founded diagnostic suspicion, “*the potential diagnostic error due to the choice of 0.70 as the fixed lower limit of normality of the ratio FEV*_*1*_*/FVC will be reduced by the extent of clinical probability of disease before performing the spirometry test*” [10]. It is significant that, even after the training day in which the reasons in favor of the LLN were presented, the percentage of those who use it did not change.

The percentage of patients with record of at least one spirometry performed within the previous year passed from adequate at the first survey (43.6%) to excellent (91.5%) at the second, while the percentage of patients with reported functional staging passed from 40% to 91.4%. Also other important parameters for the clinical staging and follow-up saw the percentage pass from unsatisfactory to good, e.g. BMI (from 33% to 69%), presence of comorbidities (from 37% to 89%), level of dyspnea (from 23.4% to 59%). Of note, regarding dyspnea, there is no standardization as yet concerning the type of scale used to measure this symptom and the heterogeneity of methods used to evaluate dyspnea is evidently a disadvantage for the appropriateness of staging and follow-up. The guidelines [10] consider only the Medical Research Council (MRC) scale, and in fact in our sample it was at the first assessment the one most used (alone or in association) and after training it remained the one most preferred.

The prescription of home oxygen therapy is governed by the pneumologist in 100% of cases and this places on pneumologists specific responsibilities that are well delineated by the guidelines [10,11]. The guidelines recommend, in fact, besides indicating the precise reference values of oxygen saturation for the prescription, to verify over time the efficacy of the chosen oxygen flows and that there is still an indication for oxygen. In our sample, the first survey showed an inadequate number of blood gas analyses reported (23.5%) but we found a considerable improvement at the second (53%), even if the percentage is still not good. In contrast, a good percentage was observed at the second survey for reported measurement of oxygen saturation, which passed from 37.3% at the first survey to 90.4% at the second. Adequate also was the percentage of centers that are organized to periodically check the suitability of continuing home oxygen therapy (27 centers out of 29, equal to 93%); of note, however, in the 6 months between the first and second survey we did not find any improvement in this latter parameter.

According to the guideline recommendations [10,11], patients’ level of severity should be graded not only by means of spirometry, but also with the use of other measurements besides those of pulse oximetry and blood gas analysis. Included in these measurements are BMI and the 6-min walking test: both these parameters saw an improvement in their recording from the first to second survey (respectively from 33% to 69% and from 15.5% to 59.2%).

Information about patients who had been treated for exacerbation by their own GP or admitted to a different ward from the outpatient clinic that usually handles them could not be directly accessed by the outpatient clinic but could only be gained from interview with the patient (often with a high level of imprecision). As a result, such information was found to be inadequate in our sample, not only at the first but also at the second survey (from 24% to 64% for exacerbation, and from 17.5% to 45% for hospitalization), testifying to the objective difficulty experienced with gathering this information. Given the importance that such information has for the grading of severity, and thus for determining appropriateness of the treatment, it is evident that the context in which the specialist operates will be inadequate until this information is available through an online network.

Reporting the prescribed long-term respiratory therapy is important for the follow-up of the patient. This report, which initially was barely adequate (40% of the sample), improved markedly at the second assessment (86%). At both surveys we observed a predictable heterogeneity of the type of pharmaceutical drugs used by the Centers. It was not the scope of this audit to evaluate the appropriateness of drug prescription of individual patients in relation to their level of severity; analyzing sensitive data of such a nature in an observational study needs in fact to be approved by the Ethics Committee of the respective Centers. However, we did observe after the training course an increased number of Centers utilizing long-acting bronchodilators (LABA or Ultra LABA or LAMA) alone or in combination with each other or together with inhaled corticosteroids (ICS), with a mean increase of 20.6% (C.I.: from +6.9 to +34.5). This finding, though merely a rough indication, combined with the significant increase seen in the reporting of long-term respiratory therapy, confirms the importance of training as a means to improve specialist appropriateness.

Finally, given that pulmonary rehabilitation (PR) is considered to be necessary at all stages of disease severity [10], the reported need for PR, which is only 23.1% of patients, and passed to 25.55% at the second survey, probably reflects a lack of offer, apparently confirmed by the low percentage of Centers that are able to offer patients a programm of pulmonary rehabilitation (45% which rose to 59% at the second assessment).

The inadequacy of the organizational context emerges strongly as regards the availability of tele-care and “specialist” home care, both of which are completely or almost completely unavailable (0% and 1%). Moreover, at the second survey there was an increase in the number of Centers completely unable to provide home care: the reason for this is unknown, but one presumes it is due to the need to reduce management costs: if this is true, it would be yet another demonstration of the inadequacy of the organizational context.

A more complex reflection is required in analyzing the responses regarding the treatment of patients in a state of acute respiratory failure. As can be seen from a comparison between the first and second assessment, after the discussion that ensued on the data collected in the first phase there was a change in how the care models active in the Centers were classified. Considering that no variation in the organization models occurred during the interval, the variation in the responses (that shifted towards a classification as internal and autonomous ICUs or sub-intensive units) should be interpreted as a greater awareness of the need to change the care models to bring them more into line with the real care needs of patients in a state of respiratory failure. As a matter of fact, knowing well both the single realities and the national context, there is no case in which an autonomous Unit exists as a structural organization, but the experiences are limited to management with ventilation in the mixed diagnostic-therapeutic reality of a Pulmonary ward.

Acute and/or acute-on-chronic respiratory diseases represent now an increasingly significant epidemiological fact, and it is indispensable that modern Pulmonary Units be equipped with beds that are reserved and adequate to receive patients in critical respiratory conditions. This would also avoid depleting the resources (instrumental and economic) of the ICUs, whose burden of patients – both incoming and outgoing - would as a result be considerably lightened. The treatment of severe respiratory failure includes as a distinctive element the use (besides pharmacological treatment) of non-invasive mechanical ventilation (NIV), assuring the patient at the same time a continuous instrumental monitoring of the vital parameters. In this sense, NIV is now in all guidelines, including the official Italian documents [10,11], established as a key part of the treatment of respiratory failure**,** and its absence constitutes a serious omission of care, which is increasingly debated in legal medicine in terms of ignorance, inability, or incapacity.

Finally, to ensure management appropriateness it is well known that the indispensable elements are: self-management, care continuity and the presence of diverse figures (not only the GP and/or specialists, but also the pharmacist and family caregivers). Concerning the possibility of educating patients about the disease and its management (“empowerment”) only 13 (which passed to 14 at the second assessment) out of 29 Centers offer this service. The procedure of jointly consulting with the GP before discharging patients (first and indispensable step for “care continuity”) is implemented in only 7 (8 at the second assessment) out of 29 Centers.

## Conclusions

At the first survey carried out, only one in three Centers on average recorded the information requested by the guidelines [10,11], and for some items even less than 1 in 6 centers recorded the information requested. Many of these percentages, however, improved substantially after the training session carried out in May 2012. It could be argued that all this effort is scarcely worthwhile, given that the lack of recording does not necessarily imply a lack of measurement and that, in fact, many – if not almost all – the parameters considered were certainly assessed as they are indispensable for formulating the diagnosis (e.g. spirometry), for staging and for the therapeutic prescription (e.g. for the prescription of oxygen therapy). It is however true that other information, whose collection is less routine in outpatient clinical practice, such as BMI, presence of comorbidities, and number of admissions for exacerbations in the preceding year, may effectively not have been “assessed”.

In any case, the lack of recording means that the patient information is not immediately available to others or “independent”, i.e. it is not “unlinked” from the professional in whose care the patient is, with the risk of perpetuating the model of episodic consultation that is not part of an organized system. Moreover the lack of availability of essential data impedes also an evaluation (internal and external) of the “appropriateness” of the steps carried out.

The Implementation forms related to the context in which the specialist operates showed far more serious gaps: in the field of smoking cessation treatment, in that of pulmonary rehabilitation, of patient education about the disease, of the continuity of care, home care and tele-care.

Our findings highlight some important points: compared to the standard represented by the guideline recommendations, both individual specialist practice and the organizational context show significant gaps. If what is in line with the recommendations can be defined as ‘appropriate’ and what is not in line as ‘inappropriate’, then a significant degree of inappropriateness exists. From the data gathered from our sample, it seems that with training and collective/individual effort the inappropriateness present in specialist practice can be resolved – and quite rapidly – but the gaps present in the organizational context (logistic-management structures) require more time to be resolved as well as a commitment on the part of other stakeholders.

Thus the question of “appropriateness” cannot be reduced to a matter concerning just the health professional or be resolved “on paper” as a matter of pharmacological therapy alone. We believe that the solution to the areas of inappropriateness highlighted depends on diverse subjects. Health professionals are certainly responsible for inappropriateness stemming from their lack of up-to-date knowledge, but Scientific Societies are responsible for the lack of standardization of instruments for clinical-functional data collection, and the National Health Service for the absence (or inadequacy) of the logistic structures required for the integrated management of chronic respiratory disease.

The editorial project ALT-BPCO shows that, with targeted training and with individual effort, specialist behavior can be modified and brought into line with what is recommended by the institutional guidelines [10,11] but it also shows that correcting the inappropriateness of the specialist is not sufficient to make the care for chronic respiratory diseases “appropriate”; for this, modification of inappropriateness “of the system” is required on the part of the NHS.

## Competing interests

The authors declare that they have no competing interests.
